# Co-expression of MARCKS and GSDMD pathway genes in tuberculous meningitis: a multi-omics analysis of blood-brain barrier disruption

**DOI:** 10.3389/fcimb.2026.1774775

**Published:** 2026-06-03

**Authors:** Mengyu Luan, Xiaoyou Chen, Yu Lu

**Affiliations:** 1 Beijing Chest Hospital, Capital Medical University, Beijing, China; 2 Beijing Ditan Hospital, Capital Medical University, Beijing, China

**Keywords:** blood-brain barrier, cytoskeleton, MARCKS, pyroptosis, tuberculous meningitis

## Abstract

**Introduction:**

Tuberculous meningitis (TBM), the most severe form of Mycobacterium tuberculosis infection, is characterized by high mortality and neurological sequelae, largely attributed to blood-brain barrier (BBB) disruption. While recent studies identified GSDMD-mediated endothelial pyroptosis as a key mechanism of inflammatory BBB damage, the full molecular landscape in TBM remains unclear.

**Methods:**

This study employed an integrated multi-omics approach, combining bulk and single-cell RNA sequencing of clinical and murine datasets with experimental validation, to identify central mediators of BBB dysfunction in TBM.

**Results:**

We identified and validated three genes – MARCKS, CD274 (PD-L1), and IL17RA – as significantly upregulated in TBM. Single-cell analysis of a murine TBM model demonstrated predominant MARCKS expression in CNS microglia, a finding further supported by elevated MARCKS protein levels in peripheral blood mononuclear cells from patients. However, validation in human TBM brain tissue remains warranted. Functional enrichment and correlation analyses positioned MARCKS at the nexus of inflammatory signaling and cytoskeletal regulation, showing strong associations with key effectors of the GSDMD pyroptosis pathway (CASP4, CD14, NINJ1). Our data further indicate a robust co‑expression pattern between MARCKS and key effectors of the GSDMD pathway (CASP5, TLR4, and CASP1), suggesting that MARCKS‑mediated cytoskeletal destabilization and GSDMD‑dependent lytic pore formation may act in concert to promote BBB disruption. Nevertheless, this proposed mechanistic interplay requires direct experimental verification.

**Discussion:**

Our findings nominate MARCKS as a novel mechanistic hub linking neuroinflammation to barrier pathology in TBM, revealing potential therapeutic targets for adjunctive barrier‑stabilizing strategies.

## 1 Introduction

Tuberculous meningitis (TBM), one of the most severe forms of extrapulmonary tuberculosis, features high mortality rates and severe neurological sequelae among survivors (Shukla et al., 2022; Barnacle et al., 2023; World Health Organization, 2025). Its clinical presentation is heterogeneous and often includes cranial nerve palsies, hemiplegia, disturbances of consciousness, and marked signs of meningeal irritation. The disease burden is particularly pronounced among pediatric populations (Navarro-Flores et al., 2022) and individuals with HIV co-infection (Huynh et al., 2022). Despite the implementation of standard anti-tuberculosis therapy, the case fatality rate in children with TBM remains as high as 19.3%, and only 36.7% of patients achieve survival without neurological sequelae (Chiang et al., 2014). A 2022 meta-analysis further demonstrated that TBM comprises 13.91% of all meningitis cases, with an in-hospital mortality rate reaching 42.12% and a markedly increased incidence of long-term neurological complications (Navarro-Flores et al., 2022). The foregoing findings underscore TBM as a formidable public health challenge, particularly in resource-limited settings. Although anti-tuberculosis chemotherapy has substantially improved overall outcomes, excessive or dysregulated host immune responses are increasingly recognized as a central driver of disease progression and tissue injury. Therefore, elucidating the molecular mechanisms linking host immunity to brain injury is essential for the development of effective host-directed therapeutic strategies (Manyelo et al., 2021; Barnacle et al., 2023).

The blood-brain barrier (BBB), a critical protective interface of the central nervous system (CNS) composed primarily of brain endothelial cells (bECs), astrocytes, and pericytes, is fundamental in maintaining cerebral homeostasis by restricting the entry of pathogens and harmful molecules into the brain parenchyma (Barnacle et al., 2023). Disruption of BBB integrity is a common pathological feature of neuroinfectious and neuroinflammatory disorders, and increased BBB permeability is widely regarded as a pivotal event precipitating neurological dysfunction and secondary immune-mediated injury (Manyelo et al., 2021; Shukla et al., 2022; Mo et al., 2024). In TBM, Mycobacterium tuberculosis may compromise BBB structure and function either through direct invasion of brain microvascular endothelial cells or via inflammation-mediated mechanisms. Infiltration of inflammatory cells, cytokine storms, and excessive production of reactive oxygen species further exacerbate endothelial hyperpermeability, thereby facilitating the propagation of meningeal inflammation and neuronal tissue damage (Barnacle et al., 2023; Navasardyan et al., 2023). Accumulating evidence indicates that BBB disruption represents not only an early pathological event in TBM but also a critical determinant of disease severity and clinical prognosis (Kumar et al., 2021; Teunissen et al., 2022).

Recently, Wei et al. demonstrated that brain endothelial GSDMD activation drives inflammatory BBB disruption (Wei et al., 2024). In this pathway, cytosolic lipopolysaccharide (LPS) is sensed by Caspase-4/11 (Caspase-4/5 in humans and Caspase-11 in mice), leading to activation of the pore-forming protein GSDMD. The resulting GSDMD pores formed in the membranes of brain bECs directly precipitate BBB disruption (Wei et al., 2024). Through the integration of knockout mouse models, single-cell transcriptomic profiling, electron microscopy, and bEC-specific viral manipulation, this study identified GSDMD-dependent pyroptosis as the core mechanism underlying LPS-induced increases in BBB permeability. Moreover, bEC-specific expression of a GSDMD-inhibitory nanobody effectively abrogated BBB damage induced by LPS or Klebsiella pneumoniae infection, thereby highlighting GSDMD as a promising therapeutic target.

In the specific context of TBM, a CNS infection, cell wall components of M. tuberculosis, such as lipoarabinomannan, may activate innate immune responses through mechanisms analogous to those triggered by LPS, potentially engaging intracellular inflammasomes and programmed cell death pathways. Nevertheless, whether the Caspase-4/11-GSDMD axis participates in TBM-associated BBB injury remains unknown. Furthermore, the upstream regulatory signals, downstream effector networks, and precise contribution of this axis to TBM pathogenesis have yet to be defined, representing a critical gap in current knowledge.

Against this backdrop, the present study seeks, for the first time, to systematically elucidate the role and mechanistic basis of the Caspase-4/11-GSDMD axis in BBB injury related to TBM. Against this backdrop, the present study addresses three key knowledge gaps. First, it systematically evaluates whether the Caspase-4/11-GSDMD axis contributes to BBB injury in TBM. Second, it identifies MARCKS as a previously unrecognized mediator linking cytoskeletal regulation to BBB pathology in this disease. Third, it examines the diagnostic potential of BBB-associated genes using independent clinical cohorts. A comprehensive, multi-modal strategy integrating bulk RNA sequencing, single-cell RNA sequencing (scRNA-seq), and *in vitro* functional assays will be employed. This approach will delineate the molecular determinants and regulatory mechanisms governing GSDMD-mediated alterations in BBB permeability during TBM pathogenesis, thereby addressing a fundamental gap in mechanistic understanding. Concurrently, it will facilitate the identification of key biomarkers closely linked to BBB disruption and allow evaluation of their diagnostic utility. These investigations are expected to establish a new theoretical framework and identify targets for early diagnosis and host-directed therapy in TBM. Importantly, although our analysis reveals co-expression relationships between MARCKS and components of the GSDMD pathway, functional synergy remains to be experimentally validated.

## 2 Methods

### 2.1 Data collection and preprocessing

**Figure 1:**
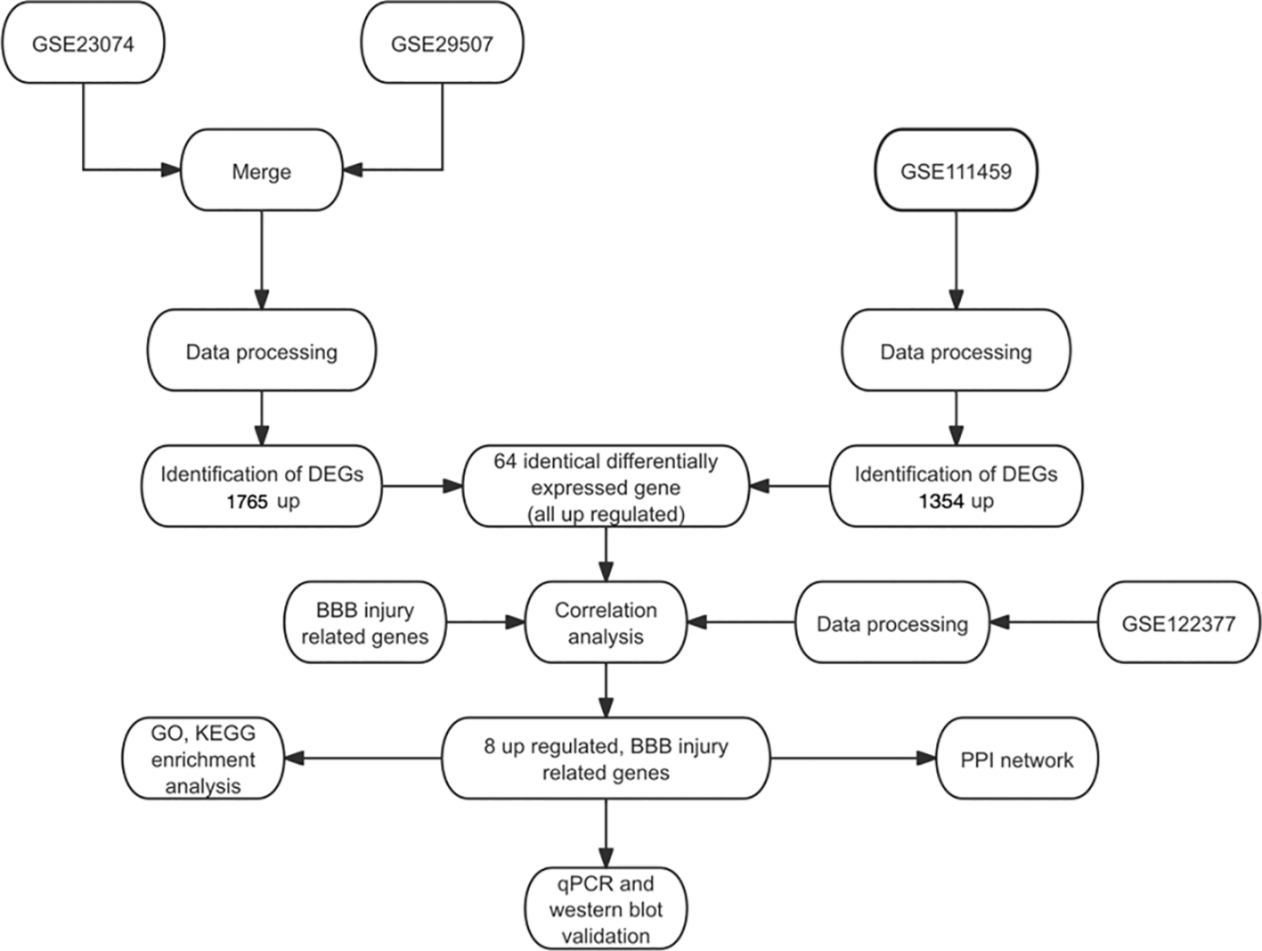
Study flowchart. (Illustrates the overall research design, including data collection, preprocessing, candidate gene screening, functional analysis, and validation.).

Publicly available transcriptome datasets related to TBM came from the Gene Expression Omnibus (GEO). The retrieved datasets included GSE23074 (five TBM patients and four head injury controls, human brain tissue), GSE29507 (five TBM patients and four head injury controls, human brain tissue), and GSE122377 (18 TBM patients and seven other infection controls, human cerebrospinal fluid). The GSE23074 and GSE29507 datasets were adjusted for batch effects via the ComBat function from “sva” in R and subsequently merged into a combined dataset (TBM_merge_data). Normalization was performed through the normalizeBetweenArrays function from “limma” in R. The GSE111459 dataset, comprising whole blood transcriptome data from 15 TBM patients, seven other infection controls, and 24 non-infection controls, was also included. The methodological workflow of this study is illustrated in **Figure 1**.

The single-cell dataset GSE199649 was selected, which contains brain tissue data from 12 TBM mouse models and 12 healthy control (HC) mice.

### 2.2 Candidate gene screening

Differentially expressed gene (DEG) analysis was conducted to compare samples from patients with TBM and HCs via “limma” in R. Analyses were performed separately in the TBM_merge_data dataset (merged brain tissue transcriptomic data) and the GSE111459 dataset (peripheral blood transcriptomic data). The screening criteria were defined as an adjusted P < 0.05 and an absolute fold change (|FC|) > 1.5, to identify genes exhibiting statistically significant differential expression in each dataset. The intersection of DEGs identified from both datasets was subsequently obtained to define a set of genes commonly dysregulated in TBM (TBM_degs). The overlap between DEGs derived from the two datasets was visualized using a Venn diagram generated with the ggvenn R package.

GSDMD-mediated blood-brain barrier (BBB) injury was delineated based on a molecular regulatory network systematically characterized by Wei et al. (2024). Spearman correlation analysis was subsequently performed to screen TBM_DEGs for genes exhibiting significant associations with at least three GSDMD-BBB-related genes. Sensitivity analyses informed the selection of an absolute correlation threshold of |r| > 0.75. Lowering the cutoff to |r| > 0.65 yielded 11 candidate genes, exceeding experimental feasibility and compromising specificity. In contrast, increasing the threshold to |r| > 0.85 identified only DAPP1, which was insufficient to capture the complexity of the GSDMD-associated regulatory network.

### 2.3 Functional enrichment analysis

Gene Ontology (GO) enrichment analysis and Kyoto Encyclopedia of Genes and Genomes (KEGG) pathway analysis were performed using clusterProfiler in R 4.12.0. An adjusted p-value < 0.05 was set as the significance threshold.

### 2.4 Single-cell transcriptome analysis

scRNA-seq data were processed via “Seurat” in R 5.1.0. Gene-cell expression matrices from different samples were integrated, and batch effects were corrected utilizing the IntegrateData function with the CCAIntegration method.

Cells were filtered according to the following criteria: (1) cells expressing fewer than 300 genes were excluded to ensure adequate transcriptional coverage; and (2) cells with mitochondrial gene expression exceeding 15% were removed to reduce potential interference from low-quality or dying cells. Following quality control, gene expression data were normalized using the NormalizeData function, and the top 2,000 highly variable genes were identified with the FindVariableFeatures function. Expression values were subsequently scaled using ScaleData, and dimensionality reduction was performed by principal component analysis (PCA) using RunPCA. Cell clustering was conducted with the FindNeighbors and FindClusters functions (resolution=0.1), based on the top 20 principal components. Nonlinear dimensionality reduction was further achieved using Uniform Manifold Approximation and Projection (UMAP) via the RunUMAP function. Finally, cell type annotation was performed via SingleR 2.8.0, with reference transcriptomic datasets employed to assign putative identities to each cell cluster (Aran et al., 2019).

### 2.5 Expression levels of candidate biomarkers and their diagnostic utility

Boxplots were generated using the ggplot2 R package to visualize and compare the expression levels of candidate biomarkers (hub genes) between TBM patients and control samples.

The diagnostic performance of candidate biomarkers was evaluated using the pROC package. Receiver operating characteristic (ROC) curves were constructed, and the area under the curve (AUC) was calculated. AUC values range from 0 to 1, with values approaching 1 indicating superior diagnostic accuracy, reflecting higher sensitivity and specificity.

To further verify the robustness of these findings, external validation was conducted using the independent dataset GSE111459.

### 2.6 Single-gene GSEA

For each identified hub gene, a rank-ordered gene list was generated based on the magnitude of Spearman correlation coefficients between the hub gene and all other genes in the transcriptomic dataset.

Single-gene Gene Set Enrichment Analysis (GSEA) was subsequently performed using the clusterProfiler R package. FEA against the GO database was carried out using the gseaGO function, while pathway enrichment analysis against the KEGG database was performed using the gseaKEGG function. This analytical framework was applied to elucidate the potential biological functions and regulatory pathways related to the hub genes.

### 2.7 Statistical analysis

All statistical analyses were conducted via R 4.4.1. The specific methods applied are detailed as follows:

Correlation analysis: Spearman correlation coefficients were calculated to evaluate relationships between gene expression levels using the cor.test() function in R.

Differential expression analysis (bulk RNA-seq): For comparisons between TBM and control groups in the merged dataset and GSE111459, the Wilcoxon rank-sum test was performed using the wilcox.test() function. A two-sided P < 0.05 was considered statistically significant.

Receiver operating characteristic (ROC) analysis: ROC curves and corresponding area under the curve (AUC) values were generated using the pROC package (version 1.18.5), with 10-fold cross-validation applied to reduce the risk of overfitting.

Experimental validation (qPCR and Western blot): Owing to the limited sample size (n=3 per group), no inferential statistical tests (e.g., Wilcoxon rank-sum test) were conducted for qPCR or Western blot data. Instead, results are presented descriptively as mean ± standard deviation (SD) to illustrate observed trends. Findings from this exploratory cohort should be interpreted with caution and require validation in larger, independent studies.

Single-gene GSEA: Gene set enrichment analysis was performed using the clusterProfiler package with default parameters, and statistical significance was determined based on a false discovery rate (FDR) < 0.05.

### 2.8 Experimental validation

#### 2.8.1 Sample collection

Peripheral whole blood samples were collected from clinically diagnosed individuals with TBM (n=3) and HCs (n=3). Every participant gave informed consent, and the study protocol was approved by the Institutional Ethics Committee.

#### 2.8.2 RNA extraction and RT-qPCR

Total RNA was extracted from whole blood utilizing TRIzol reagent as per the manufacturer’s instructions. RNA concentration and integrity were assessed via a NanoDrop spectrophotometer and agarose gel electrophoresis. Complementary DNA (cDNA) was synthesized utilizing a PrimeScript RT reagent kit. Quantitative real-time PCR (qPCR) was performed on a QuantStudio 5 Real-Time PCR System via SYBR Green Master Mix. GAPDH was the internal reference gene, and relative gene expression levels were calculated via the 2^−ΔΔCt approach. Primer sequences for all eight target RNAs and the internal reference gene (GAPDH) are provided in **Supplementary Material 1**.

#### 2.8.3 Protein extraction and Western blotting

Peripheral blood mononuclear cells (PBMCs) were isolated from whole blood by Ficoll-Paque density gradient centrifugation. Total protein was extracted using RIPA lysis buffer supplemented with protease and phosphatase inhibitors, and protein concentrations were determined by BCA assay. Equal amounts of protein (30-50 μg) were separated by SDS-PAGE and transferred onto PVDF membranes. Following blocking with 5% skim milk, membranes were incubated overnight at 4 °C with target-specific primary antibodies, followed by incubation with HRP-conjugated secondary antibodies. MARCKS protein bands were faint in control samples, while GAPDH loading control bands showed strong, uniform signal across all lanes. Blots were incubated with ECL substrate for 5 min prior to imaging. Optimal exposure time was determined to be 30 s; longer exposures (60–120 s) were tested but resulted in significant background elevation without improving the visibility of control MARCKS bands. This exposure condition was selected to ensure GAPDH bands remained within the linear detection range while maximizing the signal-to-noise ratio for MARCKS. Densitometric analysis was performed using ImageJ software, with β-actin serving as the loading control. Detailed information on antibodies, including source, host species, catalog numbers, dilution ratios, and blocking conditions, is provided in **Supplementary Material 1**.

#### 2.8.4 Statistical analysis

For the experimental validation cohort (qPCR and Western blot; n=3 per group), no inferential statistical analyses (e.g., Wilcoxon rank-sum test or t-test) were performed due to the limited sample size. Results are therefore presented descriptively as individual values alongside the mean ± standard deviation (SD) to illustrate observed trends.

## 3 Results

### 3.1 Identification of BBB-related candidate genes in TBM

**Figure 2:**
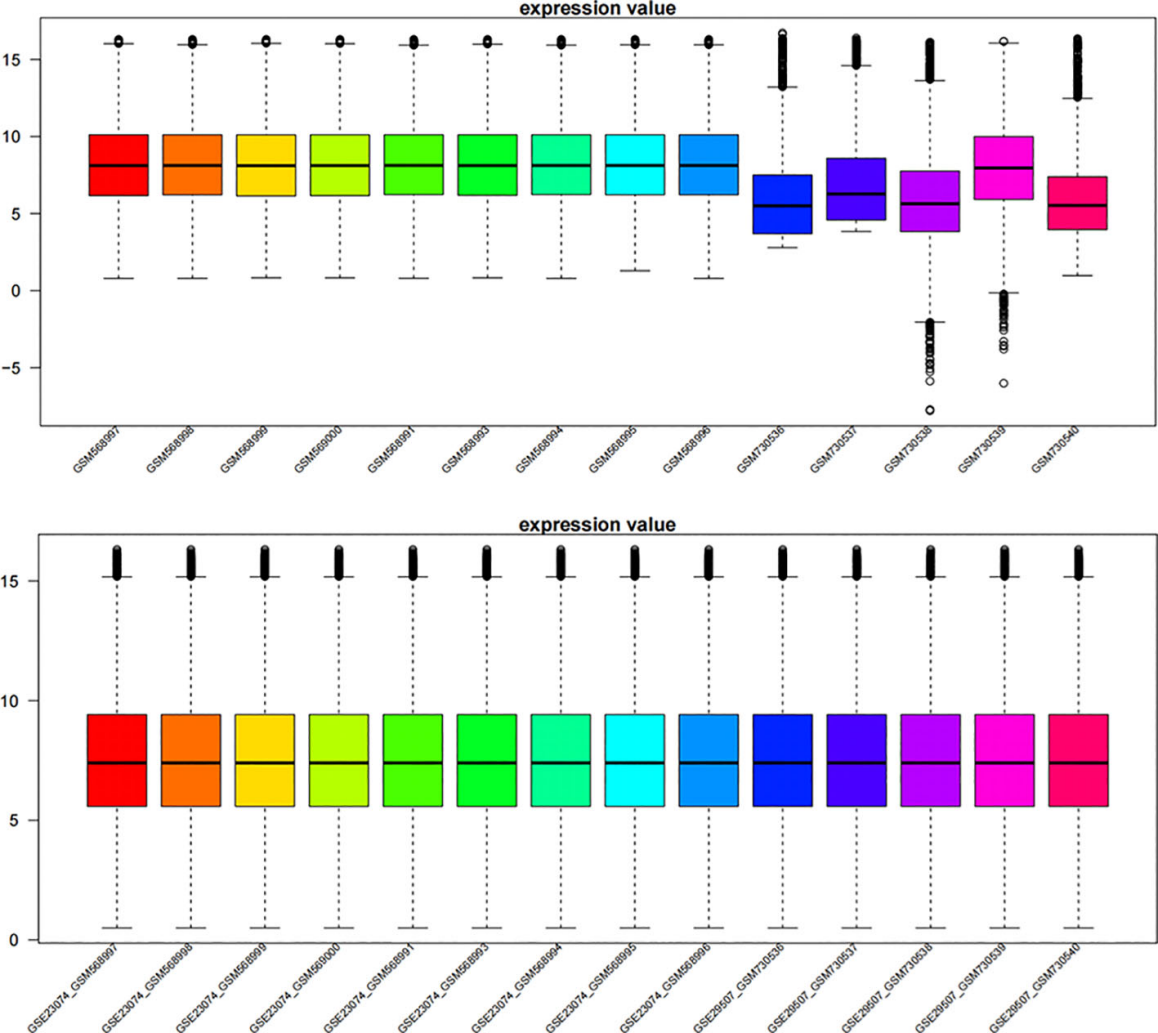
Boxplots of gene expression distribution before and after batch effect correction. (Compares expression distributions for the merged GSE23074 and GSE29507 datasets.).

**Figure 3:**
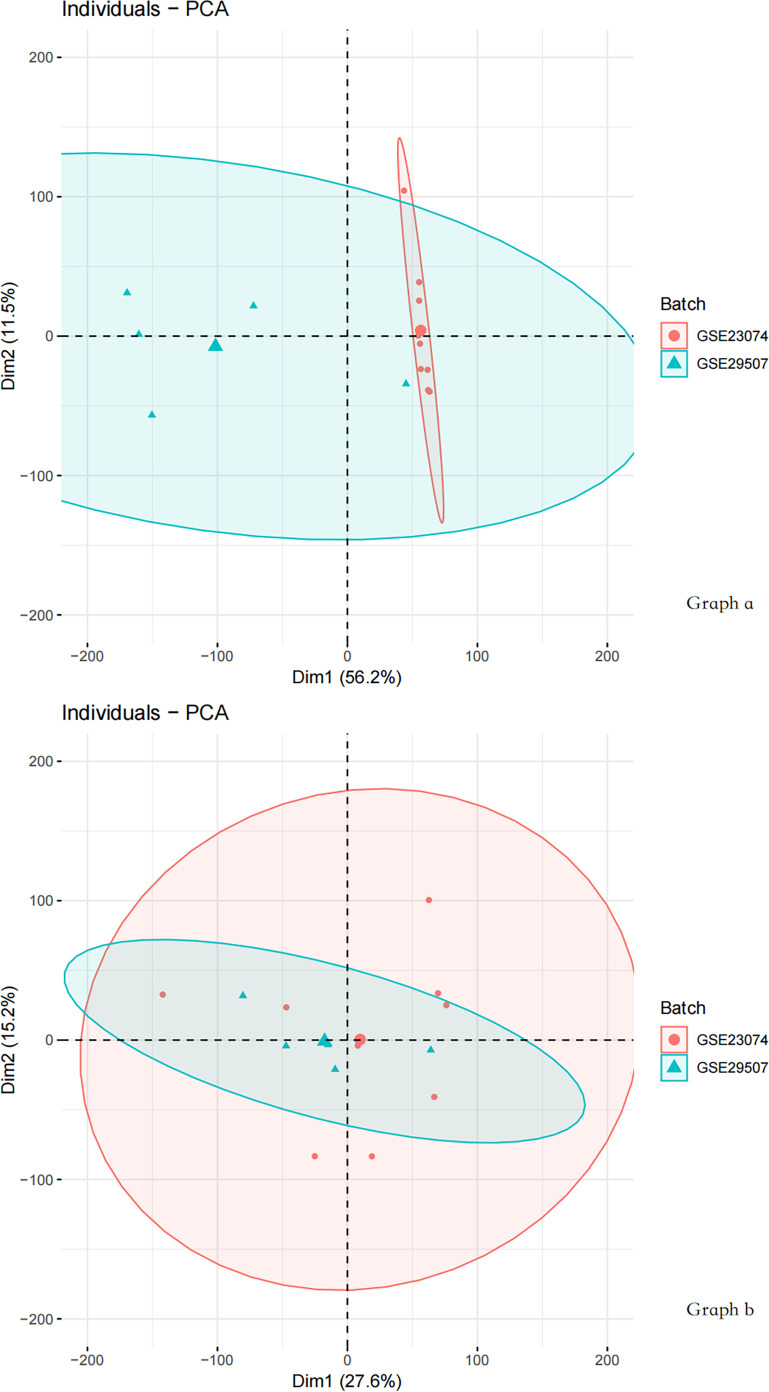
Principal component analysis (PCA) plot demonstrating batch effect correction. (Shows sample distribution and the removal of batch effects.).

Batch effects between the TBM datasets GSE23074 and GSE29507 were corrected using the sva package, and the datasets were subsequently merged to generate a combined transcriptomic dataset. Distribution boxplots (**Figure 2**) were used to compare gene expression profiles before and after batch correction, while PCA plots (**Figure 3**) were employed to assess low-dimensional feature distributions. Both approaches demonstrated effective removal of batch effects across the TBM datasets. In theory, the merged dataset comprised 18 samples, including five TBM cases and four controls from GSE23074, as well as five TBM cases and 4 controls from GSE29507. After the exclusion of duplicated samples, 14 unique samples were retained for visualization in the boxplots shown in **Figure 2**. For external validation, the independent microarray dataset GSE122377, consisting of 18 TBM cases and seven cases of other infectious etiologies, was employed.

**Figure 4:**
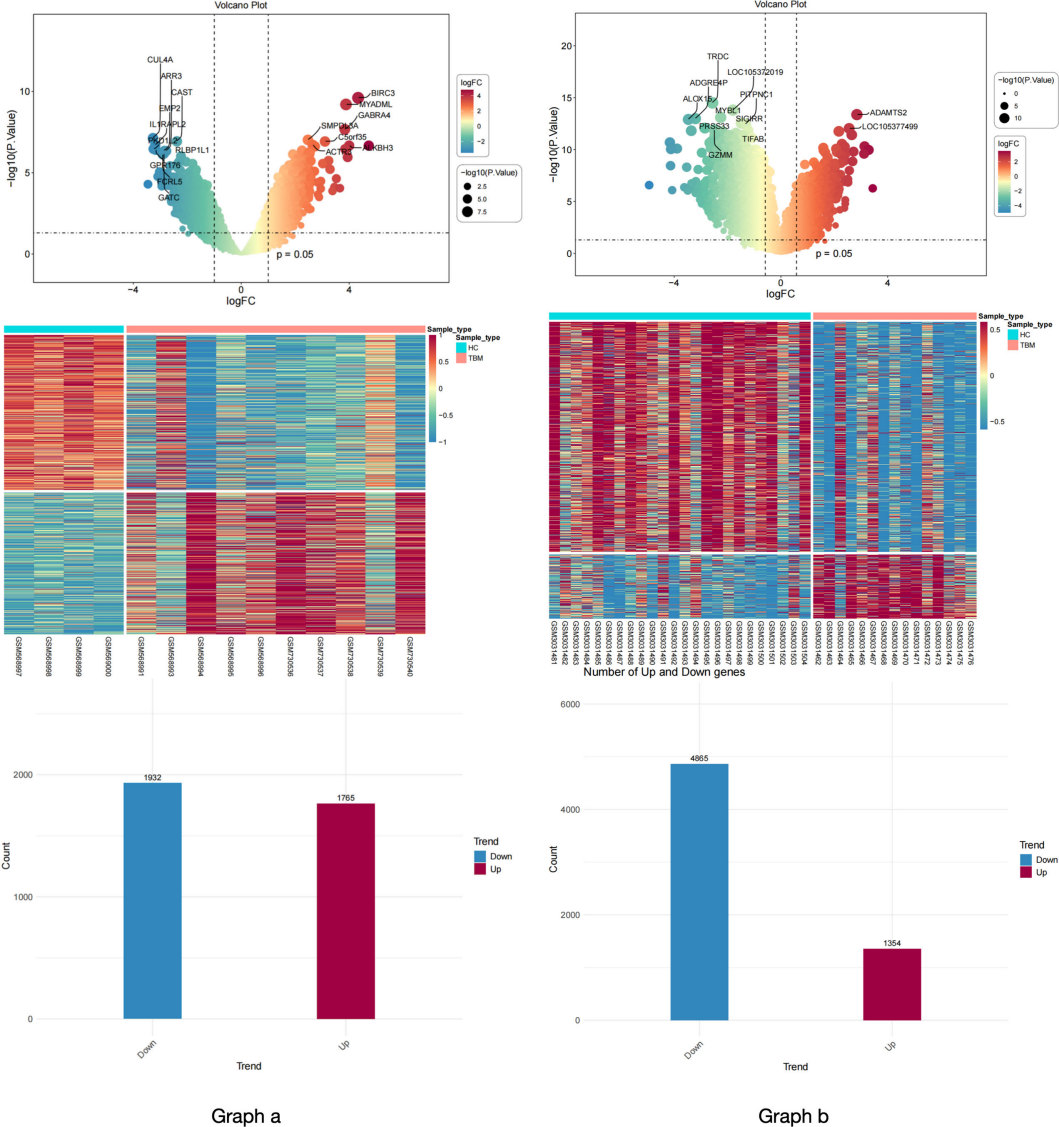
Differential expression gene (DEG) analysis results. **(a)** DEGs in the merged dataset; **(b)** DEGs in the GSE111459 dataset.).

**Figure 5:**
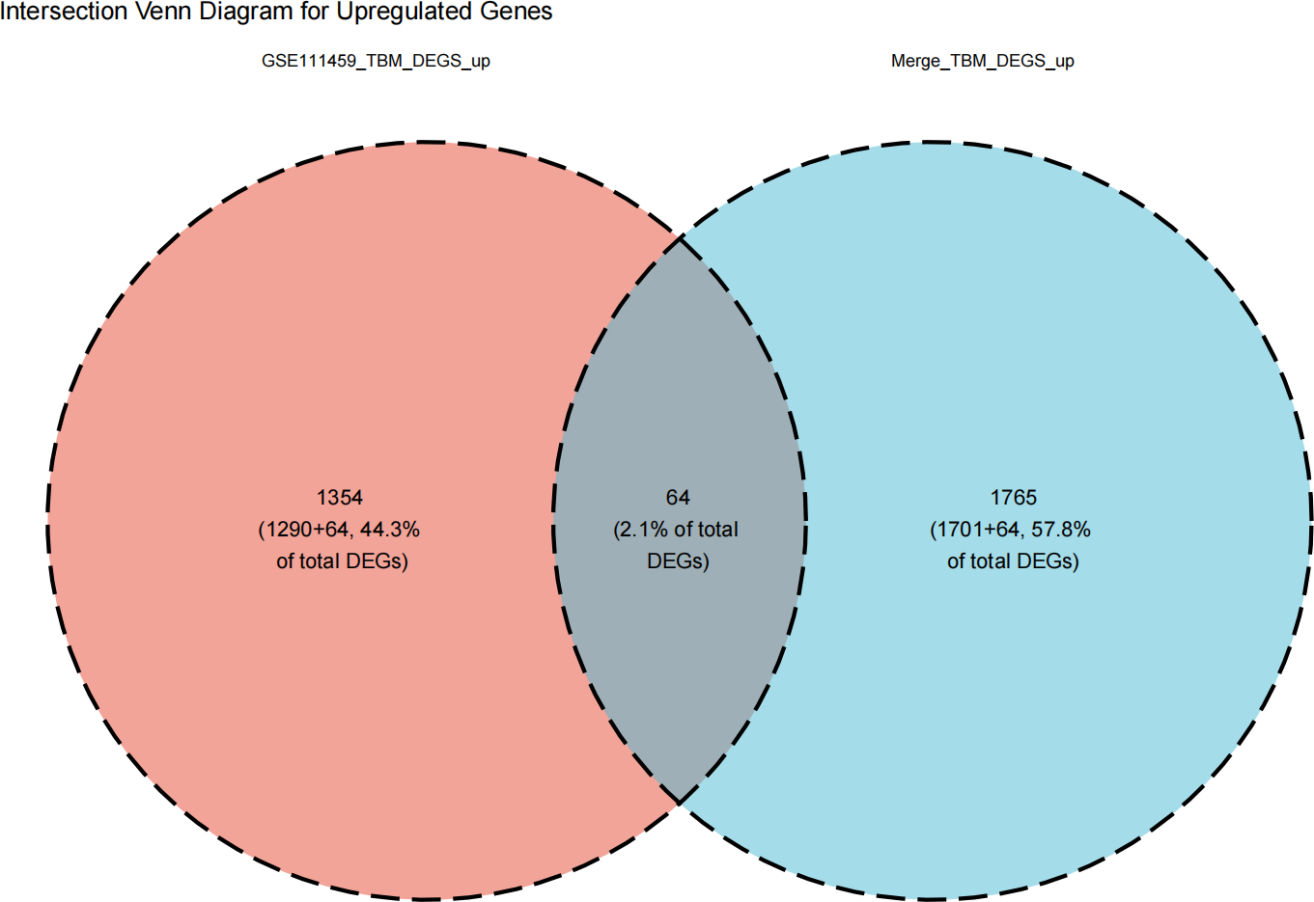
Venn diagram of overlapping differentially expressed genes. (Displays the 64 commonly upregulated genes in both brain tissue and peripheral blood datasets.).

Differential expression analysis was conducted on both the merged dataset and the GSE111459 dataset using a threshold of |log_2_ FC| > 0.585 and P < 0.05, yielding distinct sets of DEGs in each dataset. In the merged dataset, 3,697 DEGs were identified, including 1,765 upregulated and 1,932 downregulated genes (**Figure 4a**). In the GSE111459 dataset, 6,219 DEGs were detected, comprising 1,354 upregulated and 4,865 downregulated genes (**Figure 4b**). Among these, 64 upregulated genes were shared between the two datasets (**Figure 5**). These overlapping DEGs were further validated using the independent dataset GSE122377. To investigate the biological relevance of the identified genes, correlation analyses were performed focusing on ten established BBB injury-related markers, CASP4, CASP5, GSDMD, TLR4, CD14, NINJ1, IL18, NLRP3, CASP1, and TJP1. By integrating these targets, we identified eight candidate genes consistently associated with BBB dysfunction in TBM: DAPP1, FCER1G, MARCKS, IL17RA, CEBPD, ACSL4, CD274, and MCTP1 (**Supplementary Material 2**). An overview of the analysis workflow is presented in **Figure 1**.

### 3.2 FEA

To comprehensively elucidate the biological functions and pathways related to the identified gene set, systematic GO and KEGG enrichment analyses were carried out on the candidate genes.

**Figure 6:**
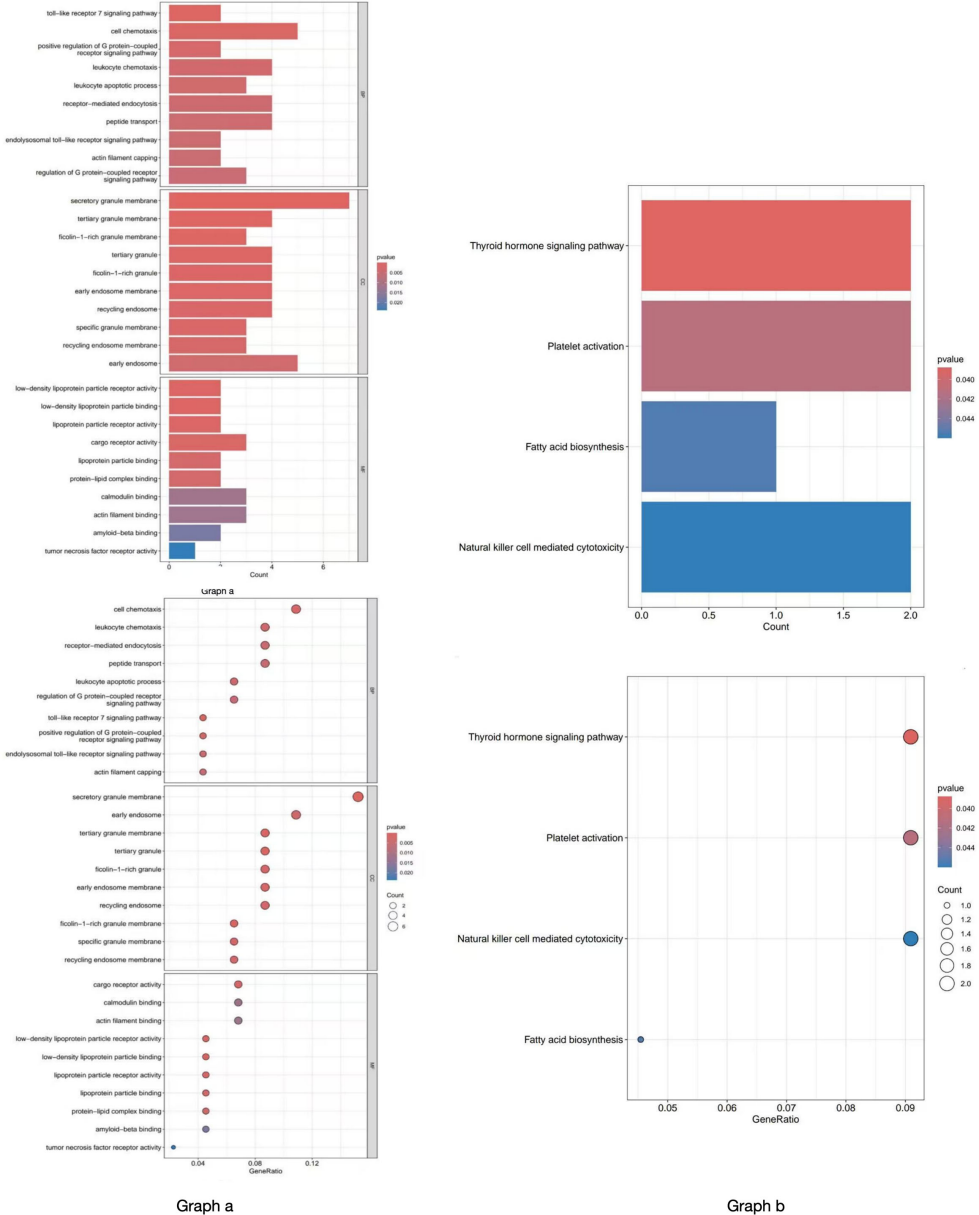
Functional enrichment analysis (FEA) results. **(a)** Gene Ontology (GO) enrichment analysis; **(b)** Kyoto Encyclopedia of Genes and Genomes (KEGG) pathway enrichment analysis.).

GO enrichment analysis yielded substantial insights across the three principal ontological domains. Within the Biological Process category, terms predominantly related to immune activation and chemotactic responses were significantly enriched, including cell chemotaxis, leukocyte chemotaxis, and the Toll-like receptor 7 signaling pathway. In addition, processes such as receptor-mediated endocytosis and the regulation of G protein-coupled receptor signaling pathways were prominently represented, indicating heightened signal transduction activity and dynamic intracellular trafficking. In the Cellular Component domain, enriched terms revealed a pronounced subcellular localization of the identified genes to intracellular granule-associated and vesicle-associated structures, including the secretory granule membrane, tertiary granule, and recycling endosome membrane. With respect to Molecular Function, significant enrichment was observed in binding- and receptor-related activities, encompassing cargo receptor activity, low-density lipoprotein particle binding, calmodulin binding, and tumor necrosis factor receptor activity (**Figure 6a**).

KEGG pathway analysis further identified several biologically relevant signaling and metabolic pathways. The thyroid hormone signaling pathway emerged as the most significantly enriched pathway. Additional enriched pathways included platelet activation, natural killer cell-mediated cytotoxicity, and fatty acid biosynthesis (**Figure 6b**).

### 3.3 Diagnostic performance of candidate biomarkers for TBM

To evaluate the diagnostic potential of the identified hub genes for TBM, ROC curve analysis was performed across datasets. The expression levels of MARCKS, CD274, and IL17RA were assessed using the R package “pROC,” and their discriminatory power was quantified using AUC.

**Figure 7:**
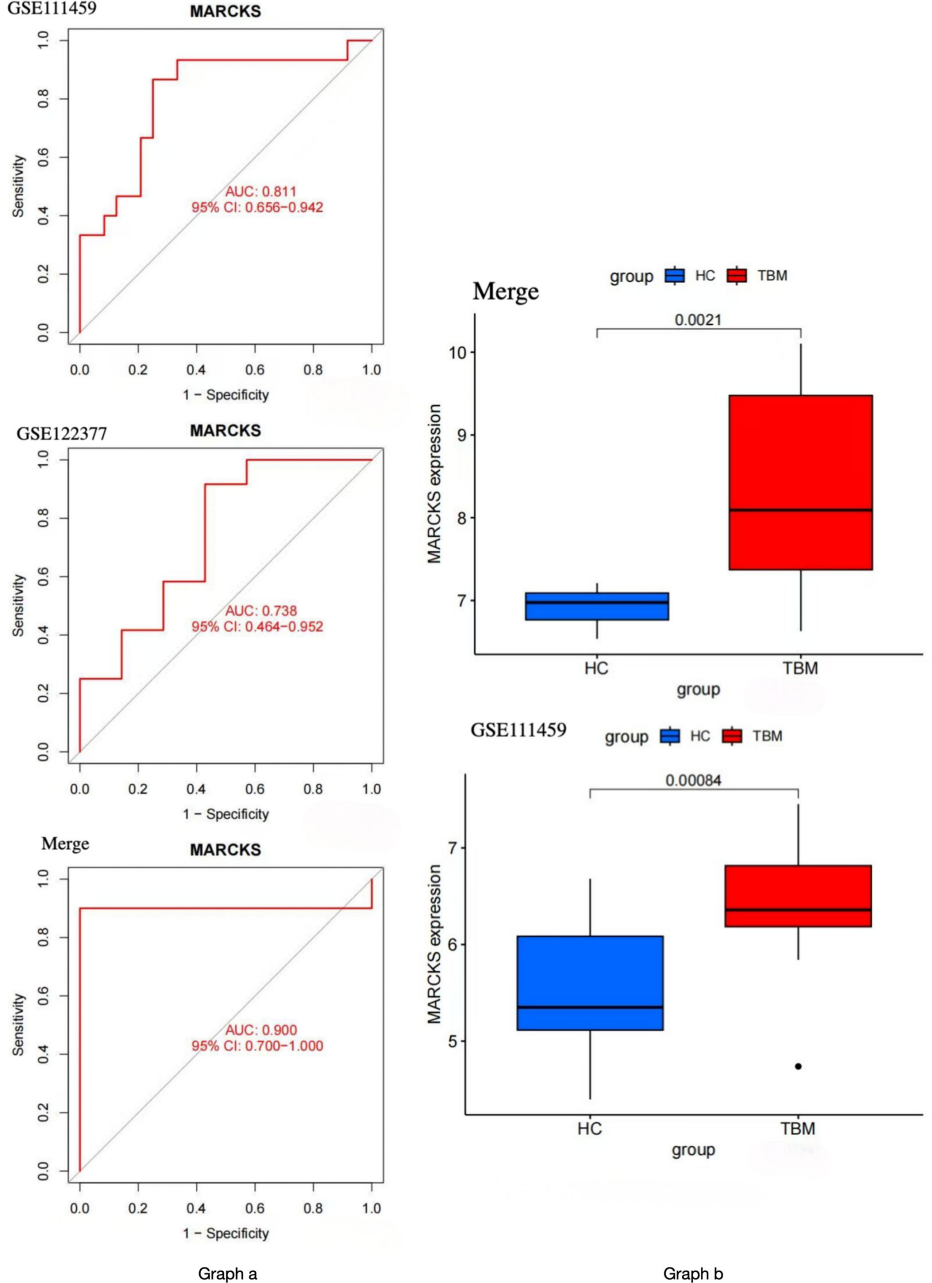
Diagnostic performance and expression level of MARCKS. **(a)** Receiver operating characteristic (ROC) curves; **(b)** Boxplots comparing expression in TBM patients vs. healthy controls (HCs).).

MARCKS exhibited robust and consistent diagnostic performance across datasets. In the merged dataset, MARCKS achieved an AUC of 0.900 (95% CI: 0.700-1.000), indicating excellent discriminative capacity. This performance was corroborated in the independent validation cohorts, with AUCs of 0.811 (95% CI: 0.656-0.942) in GSE122377 and 0.738 (95% CI: 0.464-0.952) in GSE111459, respectively (**Figure 7a**). Concordantly, MARCKS expression was significantly upregulated in the TBM group compared with HC, as illustrated by box plots from both the merged dataset and GSE111459 (**Figure 7b**).

**Figure 8:**
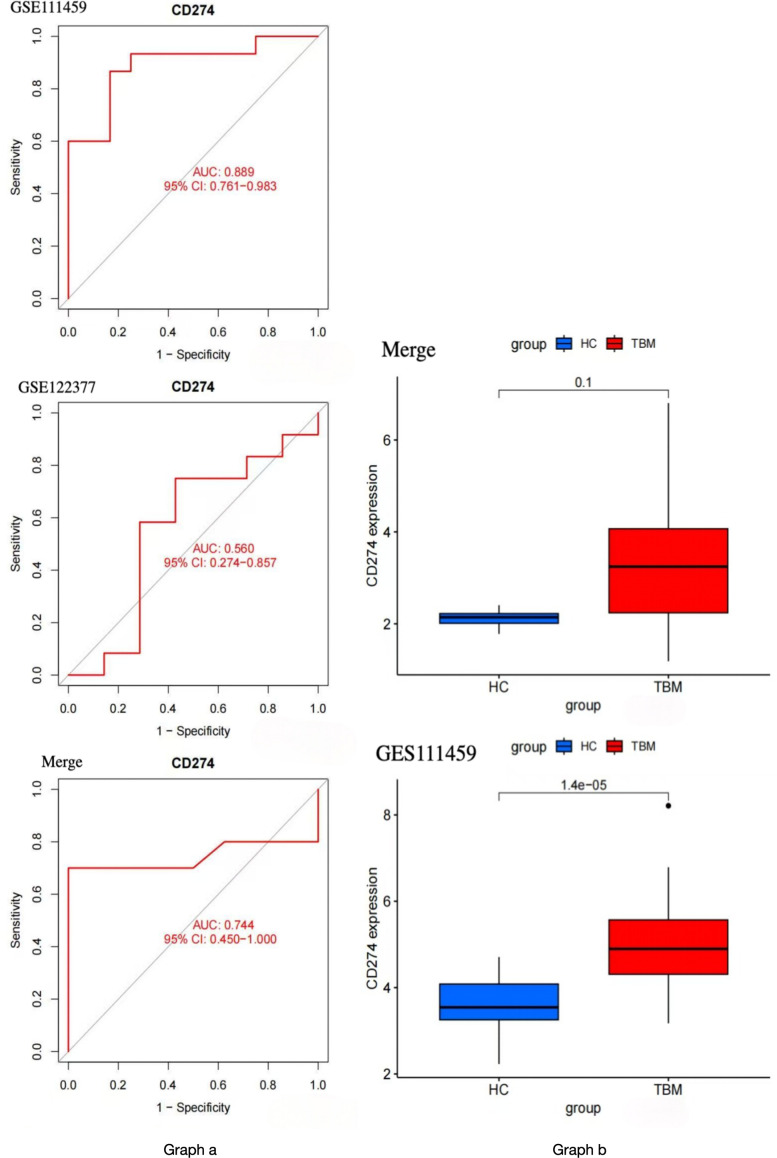
Diagnostic performance and expression level of CD274. **(a)** ROC curves; **(b)** Boxplots comparing expression in TBM patients vs. HCs.).

Similarly, CD274 demonstrated moderate diagnostic performance, with an AUC of 0.889 (95% CI: 0.761-0.983) in the merged dataset, and AUCs of 0.744 in GSE122377 and 0.741 in GSE111459 (**Figure 8a**). In terms of expression, a trend toward increased CD274 levels was observed in TBM patients compared with healthy controls (HC) in the merged brain tissue dataset (p=0.1), whereas a significant upregulation was detected in the peripheral blood dataset GSE111459 (p=1.4 × 10^-5^), as illustrated in the corresponding box plots (**Figure 8b**).

**Figure 9:**
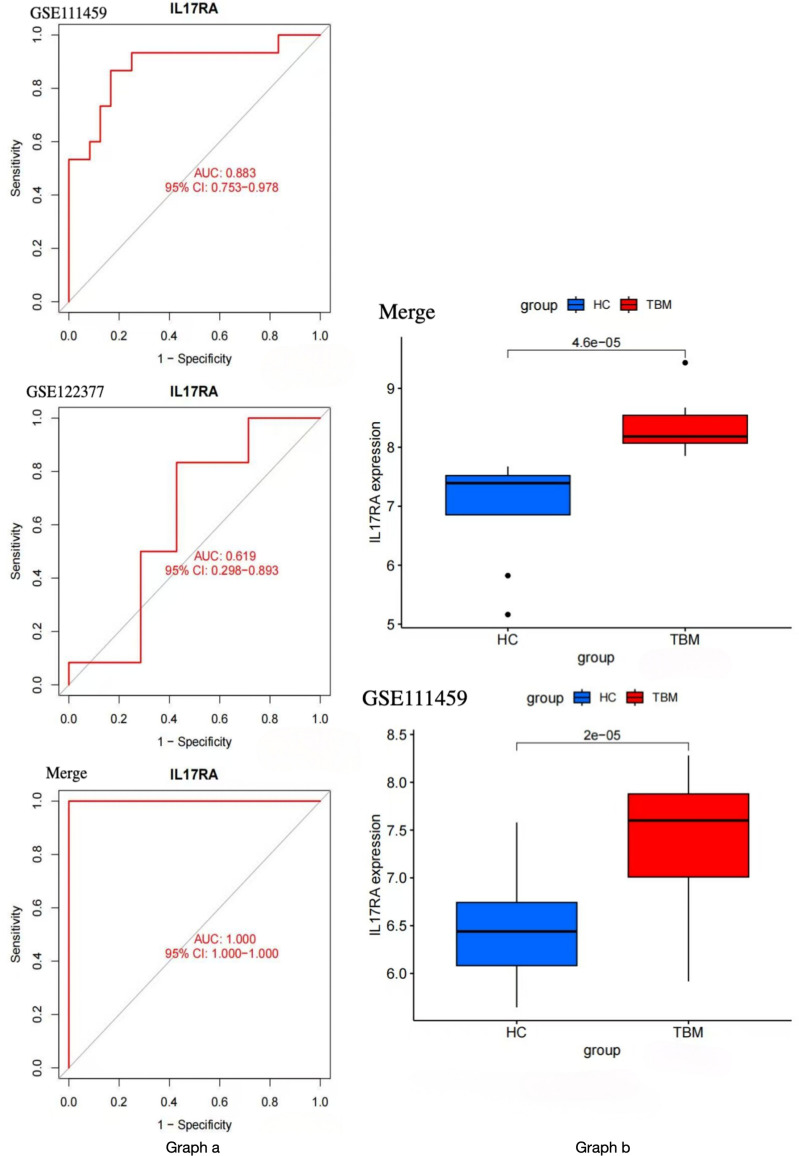
Diagnostic performance and expression level of IL17RA. **(a)** ROC curves; **(b)** Boxplots comparing expression in TBM patients vs. HCs.).

IL17RA achieved an AUC of 1.000 (95% CI: 1.000-1.000) in the combined analysis. However, this perfect discrimination likely reflects overestimation due to the small sample size and complete separation of expression values between TBM patients and healthy controls in this dataset (see **Supplementary Material 3**), and should therefore be interpreted with caution. In the validation cohorts, the AUC values were 0.800 for GSE122377 and 0.619 for GSE111459, respectively (**Figure 9a**). Consistent with these findings, IL17RA expression was elevated in TBM patients relative to HC in both the merged dataset and GSE111459 (**Figure 9b**).

### 3.4 FEA of hub genes via single-gene GSEA

To elucidate the potential biological functions and regulatory pathways related to the hub genes (MARCKS, CD274, and IL17RA) in TBM, single-gene GSEA was performed using genome-wide expression profiles ranked according to their Spearman correlation coefficients with each hub gene.

Notably, GSEA revealed a highly convergent enrichment pattern across all three hub genes. Both GO and KEGG analyses consistently identified ribosome-related pathways as the most significantly enriched categories. These included GO terms such as ribosomal subunit, cytosolic ribosome, structural constituent of ribosome, ribosome biogenesis, and rRNA metabolic process, as well as the KEGG pathways Ribosome and Ribosome biogenesis in eukaryotes. This shared enrichment pattern suggests that MARCKS, CD274, and IL17RA expression is closely related to global alterations in translational machinery and cellular biosynthetic activity in TBM.

**Figure 10:**
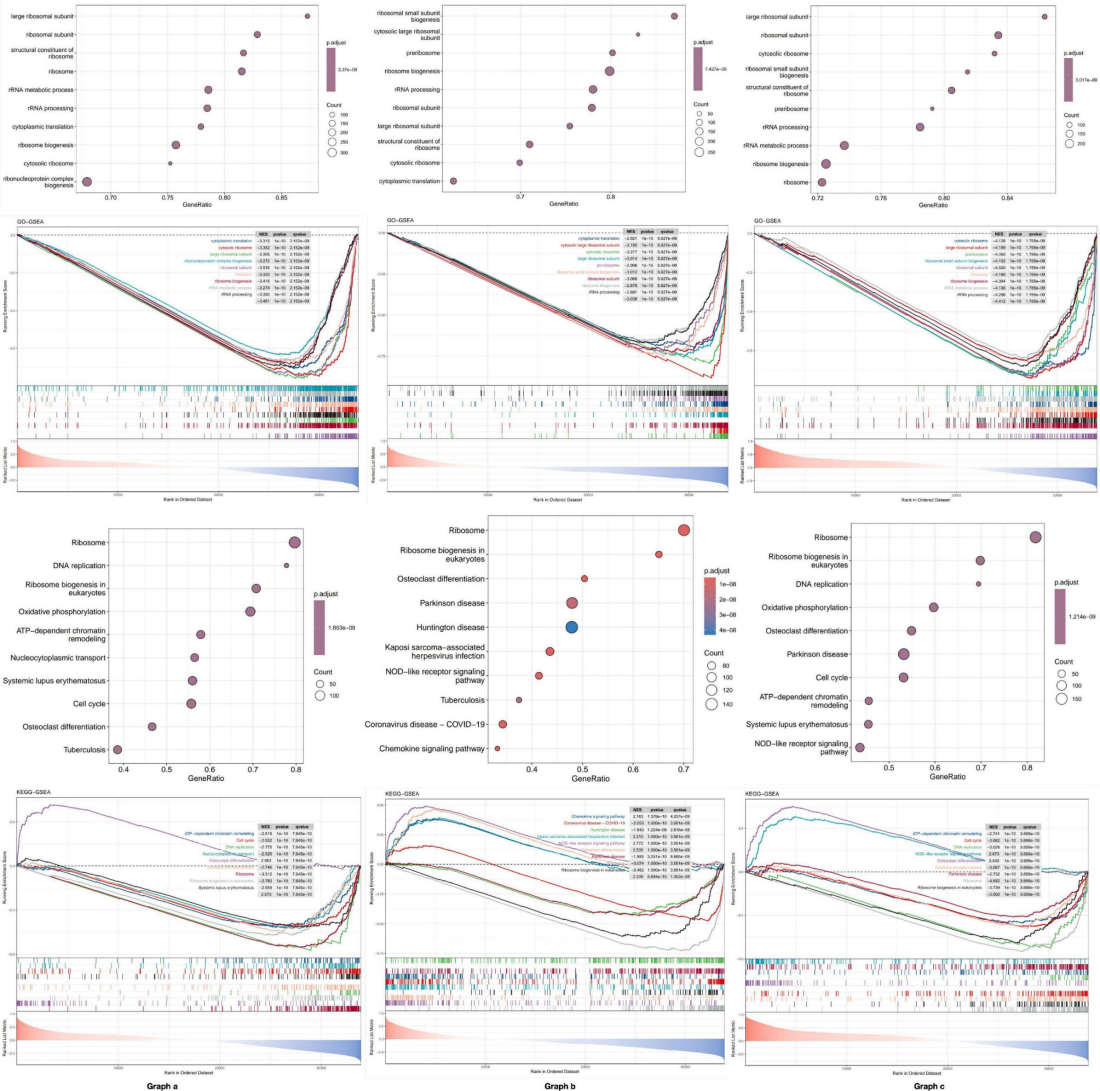
Single-gene gene set enrichment analysis (GSEA). **(a)** MARCKS; **(b)** CD274; **(c)** IL17RA.).

Beyond this common molecular signature, each hub gene also exhibited distinct pathway enrichments. MARCKS uniquely showed significant enrichment in the KEGG Tuberculosis pathway, directly linking its correlated gene expression profile to host responses against Mycobacterium tuberculosis infection (**Figure 10a**). CD274 was prominently related to immune and inflammatory signaling pathways, including the NOD-like receptor signaling pathway, Chemokine signaling pathway, and Tuberculosis, consistent with its established role in immune modulation (**Figure 10b**). In contrast, IL17RA was preferentially enriched in pathways related to cell proliferation and energy metabolism, such as DNA replication, cell cycle, and oxidative phosphorylation (**Figure 10c**).

### 3.5 Single-cell transcriptome analysis suggests microglia as the source of MARCKS

**Figure 11:**
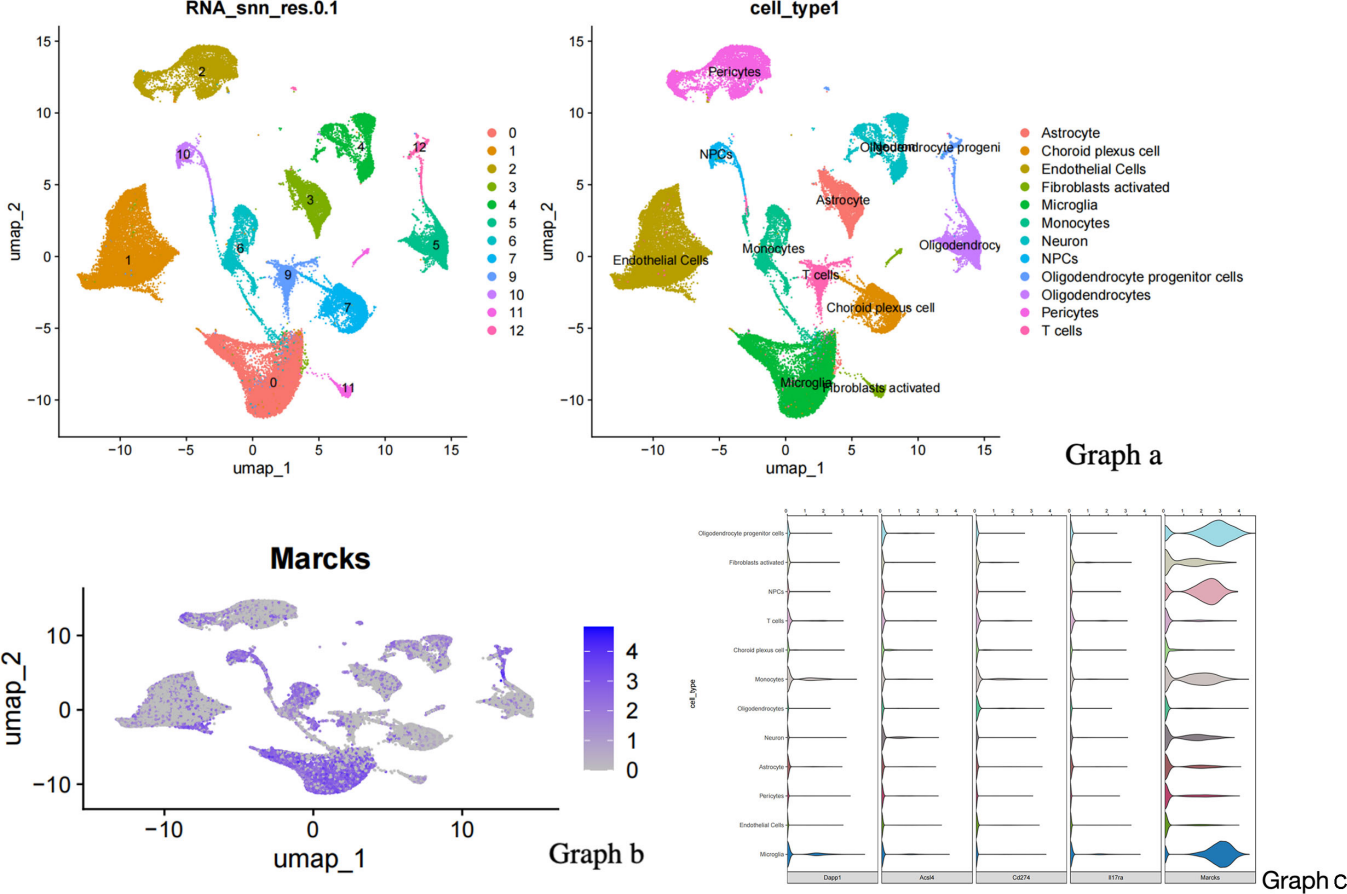
Single-cell transcriptomic analysis revealing cell-type-specific distribution of candidate genes in a murine TBM model. **(a)** UMAP plot showing cellclustering; **(b, c)**. Expression patterns of MARCKS, CD274, and IL17RA across major brain cell types.).

To investigate the potential cellular origin of the core gene MARCKS within the CNS, a publicly available scRNA-seq dataset (GSE199649) was analyzed. Notably, this dataset is derived from mouse brain tissue; therefore, the findings reflect murine gene expression patterns and may not fully recapitulate human TBM. The dataset comprised brain tissue samples from 12 murine TBM models and 12 normal control mice. Following rigorous quality control, the dataset retained for downstream analysis included 53,602 cells and 21,096 expressed genes. Based on established single-cell marker genes, twelve major cell types were identified: microglia (26.25%), endothelial cells (23.12%), pericytes (10.59%), astrocytes (7.07%), neurons (6.22%), oligodendrocytes (6.04%), monocytes (5.90%), choroid plexus cells (5.53%), T cells (3.81%), neural progenitor cells (NPCs; 2.31%), activated fibroblasts (1.65%), and oligodendrocyte precursor cells (1.53%). The expression patterns of murine homologs of the candidate genes were subsequently examined across these cell populations (**Figure 11a**).

The results demonstrated that MARCKS expression was broadly distributed across multiple brain cell types, with the highest abundance observed in microglia, followed by monocytes, NPCs, and oligodendrocyte precursor cells. Notably, appreciable expression signals were also detected in neurons, astrocytes, and oligodendrocytes. In contrast, CD274 (PD-L1) exhibited overall low expression with scattered positivity, tending to be relatively enriched in endothelial cells, microglia, and monocytes. IL17RA expression was widespread but generally low, with comparatively higher expression observed in endothelial cells, astrocytes, and activated fibroblasts (**Figures 11b, c**). These results delineate the cell-type-specific distribution of candidate genes across major brain cell populations at single-cell resolution in a murine TBM model. Whether these patterns are conserved in human TBM remains to be determined.

### 3.6 Validation of candidate genes by quantitative PCR

To experimentally validate the bioinformatic predictions, total RNA was extracted from peripheral whole-blood samples obtained from three TBM patients and three HCs. Reverse transcription followed by qPCR was performed to assess the expression levels of the eight candidate genes (DAPP1, FCER1G, MARCKS, IL17RA, CEBPD, ACSL4, CD274, and MCTP1), with GAPDH serving as the internal reference gene.

Quantitative analysis of the exploratory validation cohort (n=3/4 per group) indicated that MARCKS, CD274, and IL17RA exhibited higher mean expression levels in TBM patients compared with healthy controls, consistent with the bioinformatic predictions (**Table 1**). In contrast, the remaining five candidate genes did not demonstrate consistent directional changes in line with predictions. Notably, three genes - DAPP1, MCTP1, and ACSL4 - showed expression patterns opposite to those anticipated. Given the limited sample size, no inferential statistical analyses were performed; these descriptive observations should be interpreted with caution and require validation in larger, independent cohorts. These results support a specific association of MARCKS, CD274, and IL17RA with TBM pathology, consistent with the initial bioinformatic prioritization of these genes (**Table 1**).

**Table 1:** Expression levels of candidate genes in peripheral blood from TBM patients and healthy controls (exploratory cohort).

Gene	Fold change (TBM-1)	Fold change (TBM-2)	Fold change (TBM-3)	Fold change (TBM-4)	Fold change (HCs-1)	Fold change (HCs-2)	Fold change (HCs-3)	Fold change (HCs-4)	Mean fold change (TBM, 2^-ΔΔCt) ± SD	Mean fold change (HCs, 2^-ΔΔCt) ± SD
DAPP1	0.24	0.46	0.70	non	0.74	1.29	0.87	1.27	0.46 ± 0.23	1.04 ± 0.28
FCER1G	0.99	2.26	2.24	2.35	4.77	0.18	0.22	0.34	1.96 ± 0.65	1.38 ± 2.26
MCTP1	0.01	0.20	0.26	0.28	0.92	1.41	0.74	non	0.19 ± 0.12	1.02 ± 0.35
ACSL4	0.03	0.10	0.07	0.11	1.17	1.29	0.88	0.98	0.08 ± 0.03	1.08 ± 0.18
IL17RA	82.07	136.58	138.45	non	2.68	0.38	1.21	non	119.03 ± 32.02	1.42 ± 1.16
CD274	0.19	20.80	12.73	18.42	4.21	1.20	0.29	non	13.04 ± 9.21	1.90 ± 2.05
MARCKS	44.39	34.24	45.30	non	3.50	1.02	0.34	non	41.31 ± 6.14	1.62 ± 1.66

Due to the small sample size (n=3 per group), no inferential statistics were performed. Data are presented descriptively as mean ± SD. Individual values are shown where available. This is an exploratory analysis; findings require validation in larger independent cohorts.

### 3.7 Protein-level validation of MARCKS expression

Based on the single-cell and transcriptional findings, and to avoid redundancy with existing literature, protein-level validation was focused on MARCKS. This focus was justified for two principal reasons: first, the upregulation of CD274 (PD-L1) in tuberculosis has been extensively documented (Trinath et al., 2012); second, the IL-17RA signaling pathway has been comprehensively investigated in tuberculosis immunity, with substantial functional evidence derived from animal models (Torrado and Cooper, 2010; Freches et al., 2013; Gopal et al., 2014). In contrast, the role of MARCKS in tuberculosis remains largely unexplored.

**Figure 12:**
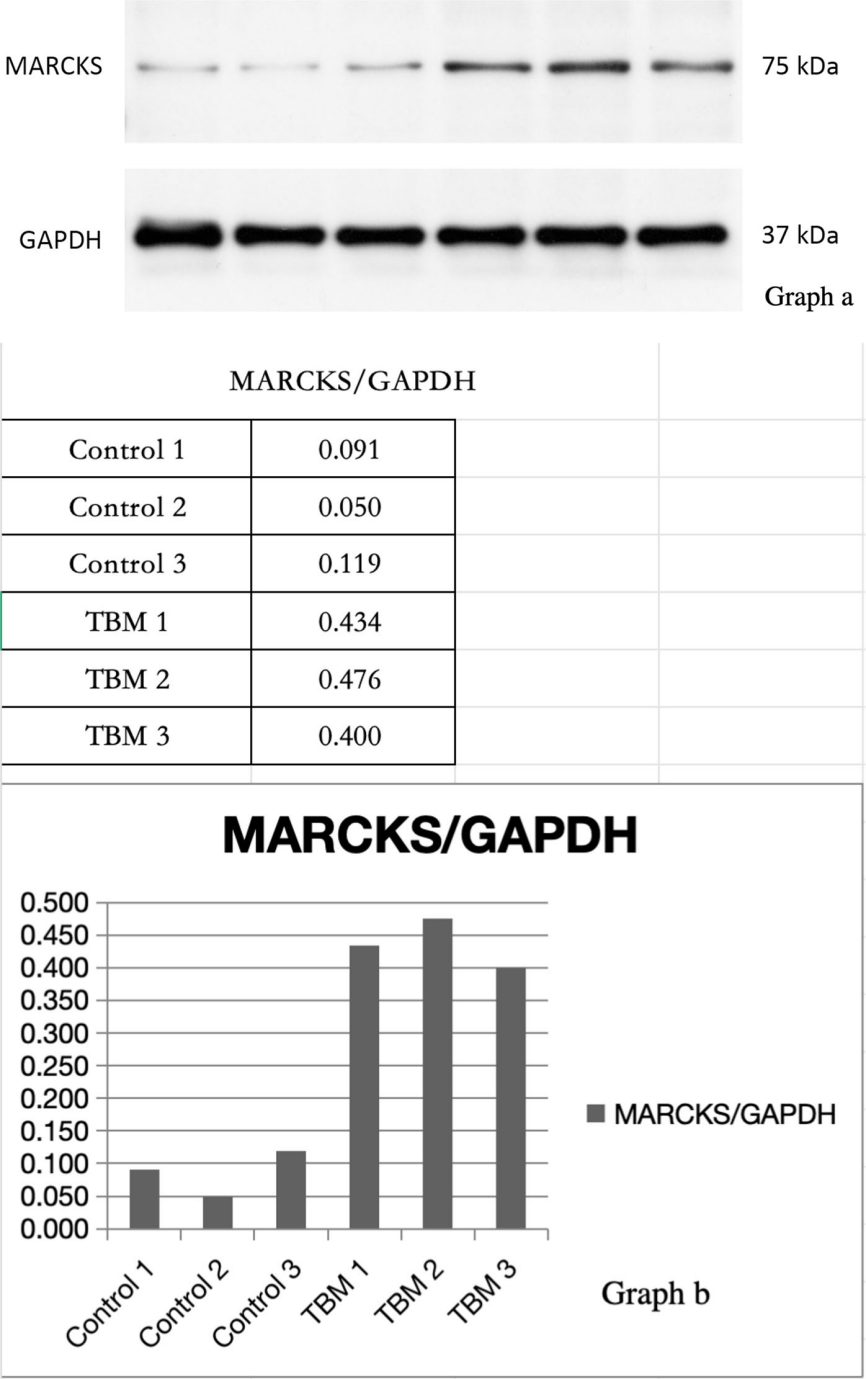
Protein-level validation of MARCKS expression. **(a)** Representative Western blot bands; **(b)** Densitometric quantification graph.).

Protein lysates were prepared from PBMCs isolated from TBM patients and HCs. Consistent with the scRNA-seq findings indicating elevated MARCKS expression in CNS microglia, increased MARCKS protein abundance was also observed in peripheral PBMCs from TBM patients. Western blot analysis of PBMCs from the same exploratory cohort (n=3 per group) revealed increased MARCKS protein band intensity in TBM patients relative to healthy controls (**Figure 12a**). Densitometric quantification showed MARCKS/GAPDH ratios of 0.091, 0.050, and 0.119 in controls, compared with 0.434, 0.476, and 0.400 in TBM patients (**Figure 12b**). The mean ratio was 0.087 ± 0.035 in controls and 0.437 ± 0.038 in TBM patients, indicating a trend toward elevated MARCKS expression in TBM. Owing to the small sample size, no statistical testing was conducted; these findings are descriptive and should be considered exploratory.

## 4 Discussion

TBM represents the most severe manifestation of Mycobacterium tuberculosis infection (Rock et al., 2008), and its pathogenesis is critically dependent on disruption of the BBB (Abbott et al., 2010; Brilha et al., 2017). In the present study, an integrated multi-omics framework was applied, combining bulk transcriptomic profiling of peripheral blood, scRNA-seq of CNS tissue, and experimental validation, to identify molecular mediators implicated in BBB dysfunction during TBM. Through this approach, eight candidate genes were identified, among which MARCKS, CD274, and IL17RA were subsequently validated and demonstrated to be significantly upregulated in TBM patients. Notably, MARCKS emerged as a novel TBM-associated factor, exhibiting pronounced enrichment in CNS microglia together with elevated expression in peripheral immune cells, suggesting its role as a central mechanistic node linking systemic inflammation to CNS barrier pathology.

The integrity of the BBB is essential for maintaining CNS homeostasis, and its breakdown constitutes a pathological hallmark of TBM (van der Flier et al., 2004), contributing to severe clinical sequelae including vasogenic edema, increased intracranial pressure, and impaired penetration of antitubercular agents into the CNS (Murthy, 2005; Costa et al., 2024; Madadi and Sohn, 2024). Traditionally, BBB disruption in TBM has been attributed to overwhelming inflammatory cytokine responses and direct invasion of mycobacteria. However, recent seminal work has identified GSDMD-mediated endothelial pyroptosis as a fundamental mechanism driving inflammatory BBB breakdown (Wei et al., 2024). In this paradigm, circulating pathogen-associated molecular patterns (PAMPs), such as LPS, are internalized via the LBP-CD14 axis, leading to activation of caspase-11 (caspase-4/5 in humans), cleavage of GSDMD, pore formation in the plasma membrane, and ultimately lytic endothelial cell death with barrier failure [provided article]. In the context of TBM, mycobacterial cell-wall components, including lipoarabinomannan, may engage analogous cytosolic sensing pathways, thereby activating the caspase-4/11-GSDMD axis in bECs and contributing to BBB injury. To systematically interrogate this pathogenic axis, a bioinformatic pipeline was implemented that integrated multiple independent GEO datasets to mitigate cohort-specific biases. This analysis identified 64 genes that were consistently upregulated across TBM blood transcriptomes. From these, eight candidate genes, DAPP1, FCER1G, MARCKS, IL17RA, CEBPD, ACSL4, CD274, and MCTP1, were prioritized based on their known involvement in endothelial permeability, immune cell migration, and inflammatory signaling, as well as their strong correlations with key components of the GSDMD-associated BBB disruption network, including CASP4, CD14, and NINJ1. The successful validation of MARCKS, CD274, and IL17RA in peripheral blood samples by quantitative PCR underscores the robustness of this integrative approach and supports the concept that peripheral immune signatures can reflect CNS-specific pathological processes, including those related to GSDMD-dependent barrier injury.

The most significant finding of our study is the elucidation of MARCKS as a crucial player. MARCKS is a cytoskeletal regulatory protein previously implicated in BBB integrity in several neurological disorders through its effects on actin organization and endothelial permeability (Jin et al., 2012; Muthusamy et al., 2022). Although MARCKS is widely recognized as a ubiquitous protein kinase C (PKC) substrate that modulates actin dynamics (Nairn and Aderem, 1992), its role in infectious diseases, and tuberculosis in particular, has remained largely unexplored. Single-cell transcriptomic analysis of a murine TBM model provided critical spatial resolution, demonstrating that Marcks (the murine homolog of human MARCKS) is predominantly expressed in microglia - the resident macrophages of the CNS and key responders to neuroinflammatory stimuli. While murine models offer valuable mechanistic insights, interspecies differences in immune responses and cell-type-specific gene expression necessitate caution when extrapolating these findings to human TBM. This localization is of substantial pathophysiological relevance, as activated microglia secrete pro-inflammatory cytokines, chemokines, and proteolytic enzymes that directly compromise endothelial integrity and BBB function (Ronaldson and Davis, 2020). Microglia occupy a central position in TBM pathogenesis by sensing M. tuberculosis, coordinating innate immune responses, and shaping inflammatory cascades within the CNS (Barnacle et al., 2023). Recent single-cell transcriptomic studies in murine models and pediatric TBM patients have demonstrated microglia-driven activation of complement pathways and inflammatory signaling networks that may exacerbate BBB dysfunction and parenchymal injury (Zhang et al., 2023b; Mo et al., 2024). These observations highlight the functional heterogeneity and specialization of microglia in TBM. The concomitant elevation of MARCKS protein levels in patient PBMCs further suggests that MARCKS upregulation represents a systemic feature of TBM-associated immune activation, potentially mirroring myeloid cell responses within the CNS compartment.

The molecular mechanisms by which MARCKS contributes to BBB disruption are multifaceted and fundamental. MARCKS acts as a critical liaison between inflammatory signals and structural failure of the barrier (Jin et al., 2012). In its unphosphorylated state, MARCKS binds to and cross-links actin filaments at the plasma membrane, promoting membrane rigidity and stability, key for maintaining endothelial tight junctions (Hartwig et al., 1992). The hyperinflammatory milieu characteristic of TBM, enriched in cytokines such as tumor necrosis factor-α and interferon-γ, robustly activates PKC isoforms (Ferro et al., 1993; Manyelo et al., 2021). PKC-mediated phosphorylation of MARCKS induces its dissociation from the plasma membrane, resulting in two interrelated consequences: loss of actin cross-linking and release of phosphatidylinositol-4,5-bisphosphate (PIP2) from sequestration by the MARCKS effector domain (Kalwa and Michel, 2011; Jin et al., 2012; Calabrese and Halpain, 2024). The liberated PIP2 subsequently activates downstream signaling molecules, including phospholipase C and actin-regulatory proteins such as cofilin, thereby promoting actin filament severing and dynamic remodeling (Wills and Hammond, 2022). This cytoskeletal destabilization facilitates actomyosin contraction, largely driven by the RhoA/ROCK signaling pathway, generating tensile forces that disrupt endothelial adherens junctions and create paracellular gaps (Mikelis et al., 2015). Collectively, these processes position MARCKS phosphorylation as a molecular switch that translates inflammatory signaling into structural BBB failure.

Beyond this canonical pathway, additional mechanistic layers may further amplify MARCKS-mediated BBB damage in TBM. First, MARCKS is closely related to vesicular trafficking and exocytosis; its phosphorylation facilitates the release of pro-inflammatory cytokines and matrix metalloproteinases (MMPs) from microglia and infiltrating monocytes (El Amri et al., 2018). Among these, MMP-9 has been strongly implicated in TBM-associated BBB breakdown, and MARCKS-driven cytoskeletal remodeling may enhance both its secretion and extracellular proteolytic activity (Chintala et al., 1998; Matsuura et al., 2000). Second, MARCKS interacts with calmodulin in a calcium-dependent manner, thereby providing a direct molecular link between inflammatory calcium signaling and cytoskeletal reorganization (Hartwig et al., 1992). Calcium overload, a frequent feature of CNS infections, may therefore function as an upstream amplifier of MARCKS dysregulation (Asmat et al., 2014; Saurav et al., 2021). Third, emerging evidence suggests that MARCKS phosphorylation influences lipid raft organization and modulates TLR4 signaling cascades, potentially intensifying innate immune responses to mycobacterial antigens and further exacerbating endothelial injury (Płóciennikowska et al., 2015; Issara-Amphorn et al., 2023). Collectively, these mechanisms position MARCKS not merely as a structural cytoskeletal regulator, but as a molecular integrator of calcium signaling, innate immune activation, and proteolytic tissue damage.

The significance of these observations is further highlighted in the context of recent advances in inflammatory BBB biology. Evidence that GSDMD activation in bECs mediates BBB disruption via pyroptosis provides a compelling parallel to the MARCKS-associated mechanisms identified in this study. In this framework, circulating LPS is internalized through the LBP-CD14 axis, leading to activation of caspase-11 (caspase-4/5 in humans), which cleaves GSDMD to form membrane pores. This process induces ionic imbalance, promotes the release of pro-inflammatory mediators, and culminates in cell lysis, with NINJ1 acting as a key executor of terminal membrane rupture. Notably, our correlation analyses revealed strong co-expression relationships between MARCKS and several critical components of this pathway, including CASP5, TLR4, CASP1, CASP4, NINJ1 and CD14 This pattern supports the hypothesis that MARCKS-mediated cytoskeletal destabilization and GSDMD-driven pyroptotic membrane disruption may act in concert to exacerbate BBB injury in TBM. However, it is important to emphasize that these associations are correlative and do not establish functional interaction. Direct experimental validation - such as co-immunoprecipitation, cellular co-localization assays, and dual-pathway inhibition studies - is required to determine whether these pathways interact mechanistically. In the context of TBM, where mycobacterial components may simultaneously activate multiple inflammatory cascades, such potential crosstalk could theoretically establish a self-amplifying cycle: GSDMD pore formation induces ionic dysregulation and cytokine release, which may activate PKC and promote MARCKS phosphorylation; in turn, MARCKS-driven cytoskeletal disorganization could enhance endothelial permeability, thereby further aggravating BBB disruption.

Beyond its canonical role in endothelial barrier regulation, the relatively high expression of MARCKS in microglia suggests an additional contribution to pro-inflammatory microglial activation. MARCKS has been implicated in macropinocytosis in macrophages (Carballo et al., 1999), a process that may initially support pathogen clearance in TBM. However, sustained stimulation could give rise to a feed-forward inflammatory loop, wherein cytokine signaling activates PKC, induces MARCKS phosphorylation, and promotes actin cytoskeletal remodeling, thereby enhancing microglial motility and cytokine release and indirectly compromising endothelial integrity. Moreover, MARCKS is essential for reactive oxygen species (ROS) generation in monocytic cells (Huber et al., 2022). Given that ROS and nitric oxide (NO) are frequently co-regulated and act synergistically in macrophage-mediated inflammation, MARCKS may also indirectly influence NO signaling through redox-sensitive pathways (Pacher et al., 2007; Casella et al., 2024). Taken together, these findings suggest that microglial MARCKS functions not only as a cytoskeletal regulator, but also as a potent amplifier of neuroinflammatory responses impacting the BBB.

The role of MARCKS also intersects with other validated immune pathways identified in this study. The upregulation of CD274 (PD-L1) is consistent with its established role in immune evasion during Mycobacterium tuberculosis infection (McCaffrey et al., 2022; Pan et al., 2022). Notably, PKC signaling has been reported to regulate PD-L1 expression, implying that the same inflammatory stimuli driving MARCKS phosphorylation may concurrently induce immunosuppressive PD-L1 expression, reflecting a paradoxical coexistence of heightened inflammation and immune suppression (Zhang et al., 2025). Similarly, the IL-17/IL-17RA axis, a potent driver of neutrophil recruitment (Huangfu et al., 2023), was strongly linked in our correlation analyses to innate immune mediators, including TLR4, CD14, NINJ1, NLRP3, and CASP1. These associations position IL17RA as an amplifier of inflammasome activation and pyroptotic signaling. Given that MARCKS is essential for neutrophil migration, it may further contribute to BBB disruption by facilitating the trafficking and pathogenic activity of IL-17-recruited neutrophils, whose extracellular traps exert profound endothelial toxicity (Sheats et al., 2015; Pieterse et al., 2017; Zhang et al., 2023a).

An intriguing finding of this study is the discrepancy in CD274 expression between brain tissue and peripheral blood. In the merged brain tissue dataset, CD274 elevation did not reach statistical significance (p=0.1), whereas a robust upregulation was observed in the peripheral blood dataset (p=1.4 × 10^-5^).

This tissue-specific pattern may have several biological explanations. First, CD274 (PD-L1) is expressed across multiple cell types, including immune cells, endothelial cells, and epithelial cells (Desimpel et al., 2025). Within the CNS, baseline CD274 expression is relatively low under homeostatic conditions, and its upregulation in TBM may be restricted to specific cell subsets (e.g., infiltrating monocytes or microglia), which are diluted in bulk tissue analyses (Manenti et al., 2022). In contrast, peripheral blood mononuclear cells - enriched for immune populations - may more readily capture systemic CD274 upregulation in response to Mycobacterium tuberculosis infection (Jurado et al., 2008; Pan et al., 2022). Second, differences in sampling time points across datasets may contribute; CD274 expression may be more prominent in peripheral blood during early immune activation, whereas CNS expression may peak later or exhibit greater heterogeneity. Third, the functional role of PD-L1 in TBM is likely context-dependent: while PD-L1 expression on peripheral immune cells may facilitate immune evasion by M. tuberculosis, its expression within the CNS may be more tightly regulated to prevent excessive immunosuppression in the brain parenchyma. These hypotheses warrant further investigation, ideally in studies incorporating paired blood and CNS samples from TBM patients.

It is important to note that the AUC of 1.000 for IL17RA in the merged dataset (GSE23074 + GSE29507) represents an overfitting artifact. As shown in **Supplementary Material 3**, IL17RA expression values exhibited complete separation between TBM patients and healthy controls, with the minimum value in the TBM group exceeding the maximum value in controls. In conjunction with the small sample size (10 TBM vs. 8 controls), this complete separation inflates the AUC and does not reflect true diagnostic performance. Therefore, the perfect AUC observed in the discovery dataset should be interpreted as a statistical artifact rather than a biologically meaningful finding.

Our study has limitations. The discovery phase relied on heterogeneous whole-blood RNA sequencing data, and although single-cell analyses were incorporated, the precise peripheral immune subsets expressing MARCKS require further immunophenotypic validation. In addition, the single-cell data were derived from a murine model rather than from human TBM patients. Although mouse models provide valuable mechanistic insights, interspecies differences in brain cell-type composition, immune responses, and gene expression profiles limit the direct translation of these findings to human TBM. Validation of MARCKS cellular distribution using human post-mortem brain tissue or single-nucleus RNA-seq from TBM patient samples would substantially enhance the translational relevance of these observations. Another important limitation is the relatively small size of the validation cohort. This limitation reflects practical challenges inherent to TBM research, including its low incidence, stringent diagnostic criteria, and the difficulty of multicenter sample acquisition. Therefore, the qPCR and Western blot results presented here are descriptive and hypothesis-generating; larger, well-characterized cohorts will be required to robustly establish the diagnostic and prognostic utility of MARCKS and related biomarkers. Finally, although we propose functional interplay between the MARCKS and GSDMD pathways based on correlative analyses and mechanistic plausibility, direct experimental validation of this interaction in TBM models remains necessary.

In conclusion, this integrative analysis provides a molecular framework for BBB disruption in TBM and identifies MARCKS, CD274, and IL17RA as key mediators. It was proposed that MARCKS functions as a central mechanistic hub, integrating inflammatory signals from the TBM microenvironment via PKC activation and translating them into both structural BBB failure through endothelial cytoskeletal collapse and amplified neuroinflammation through microglial activation. Its co-expression with components of the recently characterized GSDMD-mediated pyroptosis pathway, supported by strong correlations with CASP5, TLR4, CASP1, CASP4, NINJ1 and CD14, suggests a more complex and multifaceted mechanism of BBB disruption than previously appreciated, wherein lytic membrane damage and cytoskeletal destabilization may act in concert. However, this hypothesis remains speculative and requires direct experimental validation in relevant TBM models. This dual functionality renders MARCKS a particularly compelling therapeutic target. Collectively, this study provides the first integrated multi-omics evidence linking MARCKS to BBB pathology in TBM, nominates it as a candidate biomarker, and offers a foundation for future host-directed barrier-stabilizing strategies. The mechanistic insights presented herein suggest novel opportunities for adjunctive barrier-stabilizing therapies, including MARCKS-inhibitory peptides such as MANS or modulation of downstream contractile signaling using ROCK inhibitors such as fasudil. Future studies employing cell-type-specific knockout models are warranted to delineate the relative contributions of endothelial versus microglial MARCKS, to experimentally validate its synergy with the GSDMD pathway, and to assess the therapeutic efficacy of targeting these pathways in experimental TBM. Ultimately, combined therapeutic strategies addressing both the lytic (GSDMD) and cytoskeletal (MARCKS) dimensions of BBB disruption may offer a more effective means of preserving barrier integrity and improving clinical outcomes in this devastating disease.

## Data Availability

The original contributions presented in the study are included in the article/**Supplementary Material**. Further inquiries can be directed to the corresponding authors.

## Supplementary material

Supplementary Material 1 Oligonucleotide primer sequences for qPCR and detailed specifications of antibodies used for Western blotting.

Supplementary Material 2 Result of Spearman Correlation Analysis.

Supplementary Material 3 Distribution of IL17RA expression in the merged discovery dataset (GSE23074 + GSE29507).
