# Supplementary material for: Co-expression of MARCKS and GSDMD pathway genes in tuberculous meningitis: a multi-omics analysis of blood-brain barrier disruption
Source: Front Cell Infect Microbiol. 2026 Jun 3;16:1774775. doi: 10.3389/fcimb.2026.1774775 (PMC13272138; doi:10.3389/fcimb.2026.1774775)
Supplement: Supplementary Material 1 — Oligonucleotide primer sequences for qPCR and detailed specifications of antibodies used for Western blotting. [file SupplementaryFile1.docx]

**Supplementary Material 1**

1. **Primer sequences used for experimental verification**

| **Primer Name** | **Sequence (5' to 3')** | **Nucleotide Length** |
| --- | --- | --- |
| **GADPH** | **Forward 5’-GAAGGTGAAGGTCGGAGTCAAC-3’; Reverse 5’-CAGAGTTAAAAGCAGCCCTGGT -3’** | **71** |
| **FCER1G** | **Forward: GCCTGCATGCCATTAACACC;**  **Reverse: AACAGGGAGGAGGAACCACT;** | **58** |
| **DAPP1** | **Forward:5-GGTTACCTCACCAAACAGGGA-3；Reverse:5-GGTTCTGGTGACATCTGGTCTT-3** | **68** |
| **MCTP1** | **Forward: AGAACCTCAACCCTGTGTGG；**  **Reverse: AGGCTGAGCCCATAAAGTCA** | **57** |
| **ACSL4** | **Forward:CATCCCTGGAGCAGATACTCT；Reverse:TCACTTAGGATTTCCCTGGTCC** | **60** |
| **CD274** | **Forward:GGCATCCAAGATACAAACTCAA；Reverse:CAGAAGTTCCAATGCTGGATTA** | **61** |
| **MARCKS** | **Forward:5’-TTGTTGAAGAAGCCAGCATGGGTG-3’；Reverse:5’-TTACCTTCACGTGGCCATTCTCCT-3’** | **77** |
| **IL17RA** | **Forward:CCAACATCACCGTGGAGACC；Reverse:GTGGCGACAGCACCCTTTAA** | **57** |
| **CEBPD** | **Forward:AGTGATTAGGGAGGGCTTTAATAG**  **Reverse:GGCGGCGTCGGGCCGGGCTCTGC** | **64** |

1. **Antibodies and Dilution Methods Used in Western Blot (WB) Assay**

| **Primary Antibody Information** | | | | | |
| --- | --- | --- | --- | --- | --- |
| **Primary Antibody Name** | **Primary Antibody Host Species** | **Manufacturer** | **Catalog Number** | **Dilution Method** | **Dilution Ratio** |
| **GAPDH** | **Mouse** | **武汉三鹰** | **60004-1-Ig** | **5% Skim Milk** | **1:10000** |
| **MARCKS** | **Rabbit** | **CST** | **#5607** | **5%BSA** | **1:500** |
|  |  |  |  |  |  |
| **Secondary Antibody Information** | | | | | |
| **Secondary Antibody Information** | | **Manufacturer** | **Catalog Number** | **Dilution Method** | **Dilution Ratio** |
| **HRP-Goat anti Rabbit** | | **ASPEN** | **AS1107** | **5% Skim Milk** | **1:10000** |
| **HRP-Goat anti Mouse** | | **ASPEN** | **AS1106** | **5% Skim Milk** | **1:10000** |
| **HRP-Rabbit anti Goat** | | **ASPEN** | **AS1108** | **5% Skim Milk** | **1:10000** |
| **HRP-Goat anti Rat** | | **ASPEN** | **AS1093** | **5% Skim Milk** | **1:10000** |
| **HRP-Rabbit anti Sheep** | | **ASPEN** | **AS1245** | **5% Skim Milk** | **1:10000** |
