# Supplementary figures and images for: Co-expression of MARCKS and GSDMD pathway genes in tuberculous meningitis: a multi-omics analysis of blood-brain barrier disruption
Source: Front Cell Infect Microbiol. 2026 Jun 3;16:1774775. doi: 10.3389/fcimb.2026.1774775 (PMC13272138; doi:10.3389/fcimb.2026.1774775)

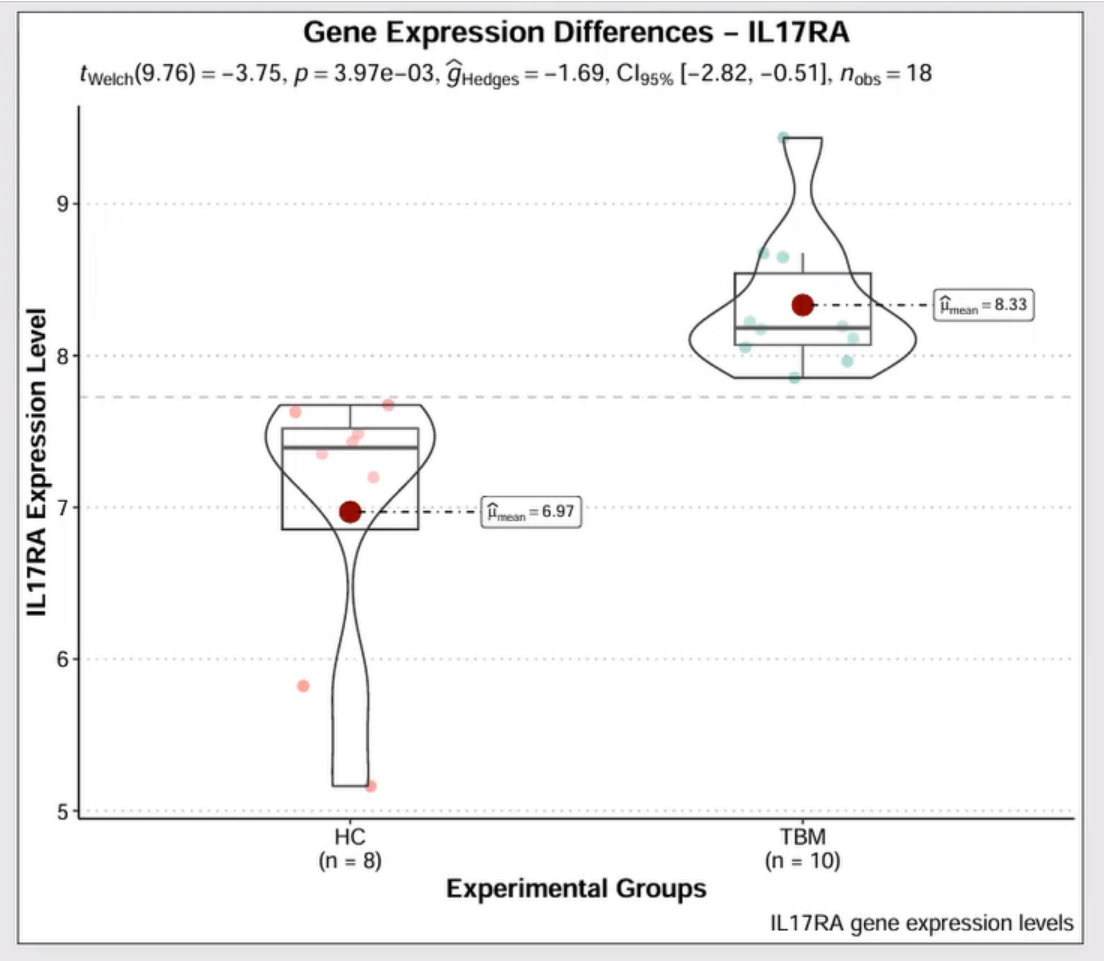

Supplement: Supplementary Material 2 — Result of Spearman Correlation Analysis. [file SupplementaryFile2.tif]
